# Potential benefits of adaptive control strategies are outweighed by costs of infrequent, but dramatically larger disease outbreaks

**DOI:** 10.1098/rsos.250598

**Published:** 2025-08-21

**Authors:** Samuel M. Smith, Colleen T. Webb, Stefan Sellman, Tom Lindström, Lindsay M. Beck-Johnson

**Affiliations:** ^1^ Graduate Degree Program in Ecology and Department of Biology, Colorado State University, Fort Collins, CO, USA; ^2^ Department of Biology, Colorado State University, Fort Collins, CO, USA; ^3^ Department of Physics, Chemistry and Biology, Linköping University, Linkoping, Sweden

**Keywords:** foot and mouth disease, emergency disease management, adaptive control, livestock disease, response delay, decision making

## Abstract

Understanding underlying transmission dynamics is necessary to effectively control an infectious disease outbreak. In the likely event that managers do not know where to target control resources because drivers of transmission are unknown, it may be desirable to tailor control strategies to a given outbreak by implementing control actions gradually in response to changes in the outbreak (adaptive) rather than all at once (fixed). Adaptive control strategies may also prevent over-reaction and thus causing unnecessary socioeconomic harm. However, it remains unclear whether the benefits of adaptive control strategies outweigh the potential of under-reacting and causing larger outbreaks. To weigh this trade-off, we used a validated national scale foot and mouth disease transmission model to compare how adaptive and fixed control strategies as well as various attributes of the control process affect outbreak size. We find that adaptive control strategies do not cost less for the vast majority of outbreaks, but infrequently result in much larger and more costly outbreaks owing to decision-making time and case reporting lags. This study emphasizes the cost of under-reacting to a disease outbreak and that minimizing decision-making time should be a key consideration when developing outbreak response guidelines.

## 1. Introduction

Controlling any infectious disease outbreak requires slowing or stopping transmission between hosts by eliminating all contact between infectious and susceptible individuals. Historically, this has been achieved through isolation, reducing the size of the susceptible pool via vaccination or depopulation (in animal systems), or a combination of these options. These control strategies are often limited by logistic and resource constraints that force decisions to be made regarding where resources should be targeted [1–5]. It can be difficult to know where to direct resources at the onset of an outbreak owing to little, if any, prior knowledge or experience. Given these uncertainties, it may be beneficial to implement control strategies that can be updated conditional on the state of the outbreak.

In this study, we will refer to adaptive control as any control strategy that is allowed to change based on any epidemiological signal from a disease outbreak. So-called adaptive control strategies have been adopted into infectious disease outbreak response guidelines for both human and animal pathogens by nations, such as the United States (US) and United Kingdom (UK), and international governing bodies, like the World Health Organization (WHO) [6–10]. For example, in the US, if there were an outbreak of the highly transmissible livestock infection, foot and mouth disease (FMD), a state-level livestock movement ban and the depopulation of infected premises (IPs) would occur immediately [7]. Then, a vaccination campaign would begin as soon as vaccines become available if the outbreak continued to spread [7]. Similarly, despite not explicitly stating an adaptive control strategy, the WHO’s Pandemic Influenza management plan suggests that countries adopt adaptive control strategies that can be targeted and scaled dependent on how quickly the outbreak is spreading and who is being affected [6]. However, many of these adaptive control strategies remain untested during real outbreaks because they were developed as improvements after control strategies became unintentionally adaptive owing to planned responses failing to deliver a sufficient response or modelling efforts identified better control strategies [11–13]. It is then necessary to understand whether adaptive control strategies can achieve desired outcomes, especially as the frequency and severity of infectious disease outbreaks increases in both humans and animals [14,15].

Policymakers and managers may favour adaptive control strategies over more fixed strategies because they could prevent over-reaction and allow for control strategies to be improved during the outbreak, thus reducing total economic burden. By promising to update the control strategy as the outbreak progresses, an adaptive control strategy can allow for a more conservative initial approach, such that fewer resources are used unnecessarily [16]. Such a response also provides managers more time to gather information regarding the outbreak’s trajectory and facilitate a more optimal distribution of resources [16]. Potentially unforeseeable logistical constraints may also change what the optimal distribution of control resources will be, so assuming a more conservative initial approach may allow managers to adjust the control strategy by identifying where the constraints arise once an outbreak has begun [17]. Therefore, adaptive control not only has the potential to save money and resources, but also improve our ability to manage an infectious disease outbreak.

Adaptive control strategies may pose several risks to controlling an outbreak despite their potential benefits. A recent body of work strongly suggests that minimizing delays during any step of the intervention process, whether that be diagnostic testing, isolating or contact tracing, greatly reduces the number of infected individuals [18–21]. Incorporating more decision-making opportunities into a control strategy then could exacerbate response delays that are already present and have been shown to result in longer and larger epidemics [22]. Further, the benefits of delaying control implementation may not outweigh the risk of allowing an outbreak beyond containment as optimal control strategies can consistently be identified very early on in an outbreak, even if projections of outbreak size are highly uncertain [23,24].

Although some of the benefits of adaptive control strategies have been demonstrated [16,17], we still lack a clear understanding of whether these benefits outweigh the potential costs of implementation. FMD outbreaks provide a valuable setting to understand these trade-offs for several reasons. First, three different control actions, movement bans, culling and vaccination, are commonly available to managers during an outbreak, so decisions must be made surrounding when to use each control action and what premises should be targeted with them. Second, the US officially plans on responding to an FMD outbreak with an adaptive control strategy [7]. Therefore, we can use the US’s FMD outbreak response strategy as a realistic starting place to evaluate how adaptive control strategies fare compared to their fixed counterparts.

Here, we consider trade-offs associated with adaptive control strategies using a validated national-scale model of FMD transmission, the United States Disease Outbreak Simulation (USDOS) [25,26]. To do so, we further developed USDOS to allow control strategies to be adaptive. We then identified several possible control strategies from the literature and conducted cattle-only FMD simulations while applying adaptive and fixed versions of these intervention strategies. Finally, we used a sensitivity analysis to understand how strongly adaptive control parameters influence FMD outbreak outcomes and whether we needed to explore other possible control strategies. The presented study provides, to our knowledge, the most in-depth evaluation of adaptive control’s ability to achieve desired outbreak outcomes to-date. Ultimately, understanding how adaptive control strategies impact infectious disease outbreaks is a key axis to improving outbreak planning and response efforts.

## 2. Methods

### 2.1. United States Disease Outbreak Simulation, v2.1

In this section, we briefly describe the version of USDOS that was available prior to this study. USDOS is a national-scale stochastic simulation model of local and long distance livestock disease spread. Local transmission was determined using a spatial kernel that represented the many modes of transmission between premises, such as fence line contact between animals on two different premises, wind moving aerosols between premises and the movement of fomites between premises (electronic supplementary material, appendix SA.1) [25–27]. Shipment-based transmission occurred via livestock shipments between premises that were modelled by the United States Animal Movement Model (USAMM; electronic supplementary material, appendix SA.2) [28,29]. Shipments predicted by USAMM were necessary to capture long-distance transmission events in USDOS.

Premises in USDOS could be general beef or dairy farms, feedlots or markets. Because the exact location and size of premises in the US are not publicly available, USDOS used 10 unique projections from the Farm Location and Animal Production Simulator (FLAPS) [30] of the number and size of premises in each county. The 10 realizations of projected premises size and locations were used to capture uncertainty regarding their exact location. Projected premises data are informed by 2012 National Agriculture Statistics Service census as well as cattle inventory surveys from July 2017 and January 2018 [25,26,30,31] (electronic supplementary material, appendix SA.3). Regardless of the type of control being implemented, all premises were given a disease status and control status at the beginning of each outbreak that was tracked throughout the simulation [25,26]. We assigned a susceptible disease status to all premises except for where we seeded an FMD infection to reflect the US livestock population’s complete susceptibility to FMD. If transmission occurred between two premises, the susceptible premises was labelled as exposed to reflect pathogen incubation times before being called infectious. After an infection period or being controlled, the premises was labelled as immune and was no longer able to be re-infected.

A premises’ control status was tracked independently of its disease status throughout each simulation and reflected what was known about a premises [25,26]. Premises began as ‘not reported’ and transitioned to either ‘reported’ (IP) if they are infectious or to ‘dangerous contact’ (DC) if the premises was at risk of infection owing to an epidemiological link to an IP. Both IP and DC premises could be controlled dependent upon the strategy being used. Premises could also be controlled in a ring around an IP, such that premises within a defined radius of the IP could be targeted for control. This ring excluded the IP itself. Once a premises was identified to be controlled, it entered a wait list of all premises that had been previously identified for control. Once the entire premises had been controlled, it was assigned an ‘effective’ status. All premises were assigned an ‘implemented’ status prior to progressing to an ‘effective’ status, but premises that were culled transition from implemented to effective immediately. There was an 11-day lag between vaccinated premises moving from implemented to effective to reflect the time required for vaccination to affect immunity (table 1).

**Table 1.** Control parameters. (Range is only listed if the parameter was included in sensitivity analysis.)

parameter	default value	range	reference
index case reporting time	15 days	2−31 days	[26]
non-index reporting time	8 days	5−25	[26]
DC reporting time	2 days	1−5	[26]
susceptible DC detectability (scaling parameter)	4	n.a.	[26]
exposed DC detectability (scaling parameter)	5	n.a.	[26]
carcass space requirements (per animal)	$1.96\;{\text{m}}^{3}$	n.a.	[26]
culling rate	240 animals premises^−1^ d^−1^	n.a.	[26]
culling effectiveness	100%	n.a.	[26]
vaccination rate	6804 animals premises^−1^ d^−1^	n.a.	[26]
vaccine doses per animal	1	n.a.	[26]
vaccine time to protection	11 days	n.a.	[26]
vaccination effectiveness	90%	n.a.	[26]
duration of immunity	183 days	n.a.	[26]
day 1 – day 5 vaccine dose availability	0 doses	n.a.	[26]
day 6 – day 13 vaccine dose availability	3 00 000 doses	n.a.	[26]
day 14 – max vaccine dose availability	5 00 000 doses wk^−1^	n.a.	[26]
vaccine dose maximum	2.5 million	n.a.	[26]
shipment ban effectiveness	75%	n.a.	[26]

### 2.2. Control in United States Disease Outbreak Simulation, v2.2.1

Control actions in USDOS consisted of movement bans, culling and vaccination. In USDOSv2.1, the implementation of these control actions was triggered immediately upon the first premises report and left in place for the duration of the outbreak simulation. We updated USDOSv2.1 to include control actions that could be dynamically applied and removed as the simulation progressed in order to implement adaptive control strategies. When adaptive control was turned on in USDOSv2.2.1, culling and vaccination were conditionally triggered in sequence (movement ban → culling → vaccination) based on outbreak thresholds using the per cent increase switch parameter that tracks the per cent increase in number of newly reported premises between two time steps (table 2; electronic supplementary material, figure SA.1). Turning on control could be further delayed by a decision-making time parameter that dictated the number of timesteps over which a per cent increase in the number of new premises reports must have been observed before control was turned on. Finally, both control actions could also be stopped depending on the number of days the outbreak had become smaller with the decrease switch (table 2). Movement bans were included in our adaptive control strategies, but cannot be implemented in response to changes in the outbreak, which means that movement bans remained in place from the time of the first report to the end of the simulation for all strategies.

**Table 2.** Adaptive control parameters. (See the electronic supplementary material, figure SA.1 for a visual depiction of how control is implemented when adaptive control is used in USDOSv2.2.1.)

parameter	definition	default strategy (control 1 $\;\overset{\;}{\to }\;$ control 2 $\;\overset{\;}{\to }\;$ control 3)	range/values	reference
switch	type of threshold used to turn control actions on and off	n.a. $\;\overset{\;}{\to }\;$ per cent increase $\;\overset{\;}{\to }\;$ per cent increase	per cent increase and number of days with no new or decreasing number of reports	expert opinion
threshold	number used to turn on a control action	n.a. $\;\overset{\;}{\to }\;$ 0% $\;\overset{\;}{\to }\;$ 10%	0–25% (per cent increase switch), 0 – 10 days (days decreasing switch)	[7] , expert opinion
action	type of control applied to premises identified to be controlled	movement ban $\;\overset{\;}{\to }\;$ cull $\;\overset{\;}{\to }\;$ vaccinate	cull, vaccinate, movement ban	[7,25,26]
target	type of premises to be targeted for control	state-level ban $\;\overset{\;}{\to }\;$ IP → DC	IP, DC, 3 km cull/vac, 10 km cull/vac	see text
priority	order in which premises are controlled	n.a. $\;\overset{\;}{\to }\;$ earliest $\;\overset{\;}{\to }\;$ earliest	earliest, smallest, largest, closest, farthest	[7,26] , expert opinion
decision-making time	the number of days where the threshold value must be observed before turning on the next control action	n.a. $\;\overset{\;}{\to }\;$ 3 days $\;\overset{\;}{\to }\;$ 3 days	0 – 10 days	[7] , expert opinion

Once control was turned on, it behaved exactly as it did in USDOSv2.1 [25,26]. In USDOSv2.1, specific premises triggered culling or vaccination based upon their infection status, risk of infection or proximity to an IP. Given this, culling and vaccination could be turned on in USDOSv2.2.1, but no culling or vaccination of premises occurred until a specific premises became reported. Premises triggered control by becoming reported if a control strategy targeted premises deemed to have had a DC or premises within a given radius of an IP, in addition to the IP (table 2).

After triggering a control action, premises entered a wait list prior to being controlled. The order of this wait list was determined by a prioritization parameter, such that premises could be controlled in the order that they entered the wait list, by their size or their proximity to an IP (only if that premises is a DC or ring culling/vaccination is on). If control was stopped owing to a lack of resources or declining number of reports, premises already on the wait list continued to be controlled until there were no more premises on the wait list. The ability to prioritize the control of premises based on their size and proximity was new to USDOSv2.2.1. All adaptive control strategies in USDOS included switch, threshold, control action, target, prioritization and decision-making time parameters, all of which are summarized in table 2 with default parameter values and ranges.

Previous versions of USDOS included several control-related parameters not addressed here because there were no updates to these parameters in USDOSv2.2.1. Of these parameters, we only included case reporting times in the sensitivity analysis described below, but all were used during simulations (table 1). Additionally, USDOS included resource constraint and control efficacy parameters. All simulations assumed movement bans were 75% effective, culling was 100% effective, and vaccines were 90% effective at blocking transmission. Please see the electronic supplementary material, appendix SA.5; Tsao *et al.* [26], and Gilbertson *et al.* [25] for more specifics about control efficacy and resource constraints in USDOSv2.2.1.

### 2.3. Simulated scenarios

We simulated the spread of FMD across the US using adaptive and fixed versions of five control strategies, resulting in 10 distinct outbreak scenarios. For each scenario, we seeded 10 outbreaks in every county in the contiguous US across 10 different FLAPS premises demography files. This design ensured that each of the 10 control strategies was simulated exactly 304 900 times, with an equal number of simulations per county. Individual simulations ran until there were zero exposed or infectious premises or until 365 days passed [26]. Every adaptive control strategy had five components: the metric that would trigger a control (switch), the threshold at which a new control would be triggered (threshold), the control that should be applied (action), the premises that this new control would target (target), the priority in which these premises would be controlled (priority), and the number of days the threshold parameter must be crossed in order to turn on control (decision-making time) (table 2). The five strategies were:

(i) state-level movement ban, IP cull, DC vaccination;
(ii) state-level movement ban, IP cull, DC cull;
(iii) state-level movement ban, IP cull, 3 km ring vaccination;
(iv) state-level movement ban, IP cull, 10 km ring vaccination; and
(v) state-level movement ban, IP cull, 3 km cull, 10 km ring vaccination.

After simulating these outbreak scenarios, we conducted three additional outbreak simulations while implementing an IP cull, DC vaccination, and state-level movement ban control strategy. We chose this strategy because it is the official United States Department of Agriculture (USDA) FMD response strategy [7]. For these simulations, we varied the decision-making time parameter (table 2) from 0 to 3 days to understand how decision-making time impacts outbreak outcomes.

### 2.4. Outbreak metrics

We considered how adaptive and fixed control strategies impacted FMD outbreak outcomes using the following commonly used metrics [25,26]:

(i) number of IPs;
(ii) outbreak duration; and
(iii) number of affected counties.

We aggregated simulation results to the county-level and calculated all metrics for the median and top 2.5% of the outbreaks to compare how adaptive and fixed control strategies affected the most common and the largest outbreaks. We calculated the metrics separately because distributions of the outbreak metrics were highly bimodal, such that a small subset of the outbreaks infected more than 10 000 premises, while the vast majority infected fewer than 1000 premises. We also considered the proportion of FMD spread attributed to local versus shipment-based transmission. This metric should indicate whether adaptive control strategies altered national-scale patterns of disease spread and was calculated by dividing the number of new infections owing to local transmission by the total number of transmission events during a simulation.

Next, we estimated the cost of each outbreak to evaluate whether delaying the implementation of control actions offered any economic benefit. We modified a previously developed cost function to estimate the cost of each outbreak under three different control scenarios [16,32]:


(2.1) $$C_{ij}=255.5\times H_{ij}+6\times V_{ij}+2160000\times D_{ij},$$

(2.2) $$C_{ij}=255.5\times I_{ij}+6\times V_{ij}+2160000\times D_{ij},$$

(2.3) $$C_{ij}=255.5\times I_{ij}+255.5\times V_{ij}+6\times V_{ij}+2160000\times D_{ij}.$$

Equation (2.1) represents the cost of an outbreak for which culling only occurred during an outbreak, such that previously infected but recovered animals were not culled. Equation (2.2) is the cost of an outbreak where all infected animals were culled. Equation (2.3) calculates the cost of an outbreak where all infected and vaccinated animals were culled. All cost calculations were strongly driven by the duration of the outbreaks because of the high cost of trade restrictions [33,34]. Cost is calculated in US dollars (USD) and is estimated for the $i^{\text{t}\text{h}}$ replicated outbreak for the $j^{\text{t}\text{h}}$ FLAPS realization. Variable H was the number of cattle culled, V is the number of cattle vaccinated, D is the duration (in days) and I is the number of infected animals for the $i^{\text{t}\text{h}}$ outbreak of the $j^{\text{t}\text{h}}$ FLAPS realization. The original equation was developed to estimate cost in pound sterling for the UK, so we modified the equation to estimate the cost in USD by using US-specific coefficients published by Schoenbaum *et al.* [33]. We also added terms to incorporate the cost of vaccination and an outbreak’s duration to account for lost agricultural sales both nationally and internationally as a function of the outbreak’s duration. Cost of culling includes the cost of the act of culling as well as indemnification [33,34]. We do not distinguish between the cost of an animal dying from FMD infection and culling the animal because FMD is rarely fatal in adult cattle [35] and animals that died as a result of infection must still be removed from the premises, the cost of which is incorporated in the culling coefficients in Equations (2.2) and (2.3). We only estimated cost for outbreaks that ended prior to 365 days because our simulations ended, regardless of outbreak size, when an outbreak reached 365 days to manage computation time. Therefore, by excluding these outbreaks, we avoided underestimating their cost as they were ongoing outbreaks. We note that these equations do not account for many other economic factors, including the number of days after the outbreak is over before trade restrictions are lifted, which means the costs presented should not be taken for exact outbreak cost estimates. Instead, these cost estimates are meant to be relative metrics by which we can compare adaptive and fixed control strategies.

To understand what could be driving cost differences between adaptive and fixed control strategies, we evaluated the probability that a state-level movement ban, IP cull, DC vaccination control strategy completes and the probability that an outbreak fades out for each control strategy. We defined outbreak fade-out to be when an outbreak that spread beyond the index infection subsided prior to reaching 5000 IPs and 365 days. This cut-off was chosen based on the natural break in the distribution of the number of IPs that USDOS predicts and has been used for previous USDOS publications [25]. We used a generalized linear model with a binomial distribution and logit link to predict both probabilities. In both models, we regressed dummy variables indicating whether or not the control sequence had completed or the outbreak had faded out by days required for decision-making and duration of the outbreak. We allowed both predicting covariates to interact, which allowed us to also assess how decision-making time impacted both probabilities.

### 2.5. Sensitivity analysis

We conducted a sensitivity analysis to determine the impact of adaptive control parameters on outbreak outcomes. We also used the results from these analyses to explore alternative adaptive control strategies as they indicate how the specific parameter values influence an outbreak. We used latin hypercube (LHC) sampling to generate 100 adaptive control parameter sets. Each parameter set was run with a state-level movement ban, IP cull and DC vaccination protocol. Like with the other outbreak scenarios presented, all farms were controlled in the order in which they were identified as being infected (e.g. earliest prioritization parameter, table 2) because prioritizing premises by their size or distance to IPs did not change how well control strategies performed (electronic supplementary material, figure SB.2). Our LHC sampling included threshold and decision-making time parameters (table 2) as well as reporting times for index, non-index and DC cases (table 1). We ensured that pairings of threshold and decision-making time parameters always resulted in culling being turned on prior to vaccination. We did not vary any disease or resource-related parameters in our sensitivity analysis as their impact on outbreak metrics has been previously evaluated [25,26]. Values and ranges of parameters used in LHC sampling are listed in tables 1 and 2.

Thresholds for the three different control switches (per cent increase in number of reports between two time steps, landfill capacity or vaccine availability and number of days with decreasing number of reports, table 2) cannot be compared to each other. To address this, we conducted a preliminary sensitivity analysis where we systematically held the control switch and control action type constant across all parameter sets and varied the remaining control parameters using the LHC sampling protocol described. This preliminary analysis revealed that outbreak metrics were very weakly influenced by control switch type. Given those preliminary results, we only considered the per cent increase switch from thereafter to reduce the dimensionality of our sensitivity analysis and facilitate interpretation.

To conduct enough simulations for the sensitivity analysis while maintaining reasonable computation times, we seeded infections in a subset of 86 counties that were selected in two steps [25,26]. First, 78 counties were selected using stratified random sampling of county characteristics and then eight additional counties were added to this sample to improve geographical reach and ensure that counties known to be important to the cattle industry were included in the sample [25–27]. County characteristics that we considered in the stratified random sampling include premises density, number of in- and out-shipments, and premises clustering values. Tsao *et al.* [26] provides a list of the 86 counties as well as an in-depth description of how the sampling was done. We seeded infections in each of the 86 counties 100 times for each of the 100 parameter sets, for a total of 860 000 simulations that were used in the sensitivity analysis.

We used linear regression to quantify the effect of each parameter set on outbreak metrics. We prefer the use of regression over other tools commonly used for sensitivity analyses because it provides greater accuracy [36] and allows us to estimate interaction coefficients. However, the relationship between these parameters and outbreak metrics are highly bimodal and often nonlinear, thereby violating regression’s assumption that the relationship between average outbreak metric value and control parameters is strictly linear. However, regression is quite robust against this assumption. We evaluated whether violating regression’s linearity assumption produced expected results by comparing results of partial-rank correlation coefficients (PRCCs) and regression analysis without interaction terms. PRCC is a useful tool for this comparison because it has the less strict assumption that there is a monotonic relationship between covariates and response variables [37]. Upon confirming a monotonic relationship was present in our data, and once we were confident that the results of the PRCC and regression analyses matched, we decided to proceed with using regression to estimate the relative effect of parameters on outbreak metrics for the rest of the sensitivity analysis (electronic supplementary material, figure B.6) [25–27].

## 3. Results

### 3.1. Effects of control on outbreak metrics

**Figure 1.**
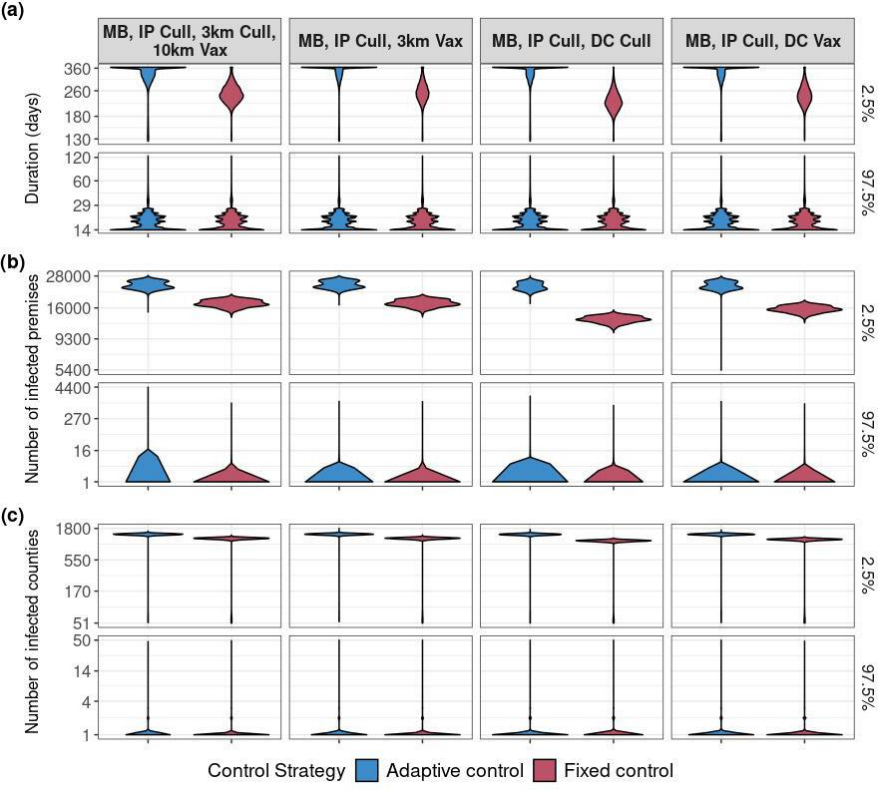
Comparison of adaptive and fixed control strategies using three different outbreak metrics. Outbreak outcomes are similar between adaptive (blue) and fixed (red) control strategies for the smallest approximately 97.5% of outbreaks (bottom facet of each panel). Each panel shows the effect fixed and adaptive control strategies on (a) outbreak duration, (b) the number of IPs, and (c) the number of infected counties. Panels are faceted horizontally such that approximately 97.5% of outbreaks are in the bottom row and the largest approximately 2.5% of outbreaks are in the top row. Control strategies are grouped by columns. Each policy includes a movement ban (MB), culling and vaccination. For adaptive control, movement bans are triggered upon the first reported premises, and culling begins the following day, while approximately 10% increase in new premises reports for 3 days triggers vaccination. The targets of these control actions vary for each control strategy, and may be one of the following: infected premises (IPs), dangerous contacts (DCs) and or all premises within a 3 km/10 km radius from an IP.

Distributions of outbreak metrics were bimodal for all control strategies (figure 1). The majority of outbreaks (89%) did not spread beyond the initial index infection or remained very small (96% below 10 IPs). Outbreak duration and the number of counties infected both followed similar patterns, where 91% of outbreaks lasted less than 30 days and IPs in only one county.

The effect of control strategies on outbreak duration, number of infected counties and number of IPs varied very little for the smallest 97.5% of outbreaks, regardless of whether the policy was implemented as adaptive or fixed (figure 1). Control strategies that targeted DCs for control performed better than policies that targeted premises by proximity, regardless of whether control actions were implemented as adaptive or fixed (figure 1). IP culling with DC culling performed the best across all outbreak metrics for both adaptive and fixed control strategies, closely followed by IP culling with DC vaccination. For adaptive control strategies, prioritizing premises for control based on their distance to an IP or size had no effect on the performance of the IP culling with DC vaccination control strategy (electronic supplementary material, figure SB.2). Prioritizing premises by distance to an IP improved the IP culling with 3 km ring vaccination control strategy, but did not change the overall pattern of which control strategy performed best (electronic supplementary material, figure SB.2). We will only consider an IP cull and DC vaccination from hereafter because it was one of the top two performing control strategies and it is the USDA’s official FMD response policy [7].

### 3.2. Fixed versus adaptive control strategies

Adaptive control performed similarly to fixed control strategies for 97.5% of outbreaks (figure 1). For the most extreme outbreaks, adaptive control strategies resulted in longer outbreaks as well as more IPs and counties than fixed control strategies (figure 1). More simulations reached the 365 day cut-off when adaptive control was applied to the outbreak, which means the outbreak never faded out (figure 1; electronic supplementary material, table SB.5).

**Figure 2.**
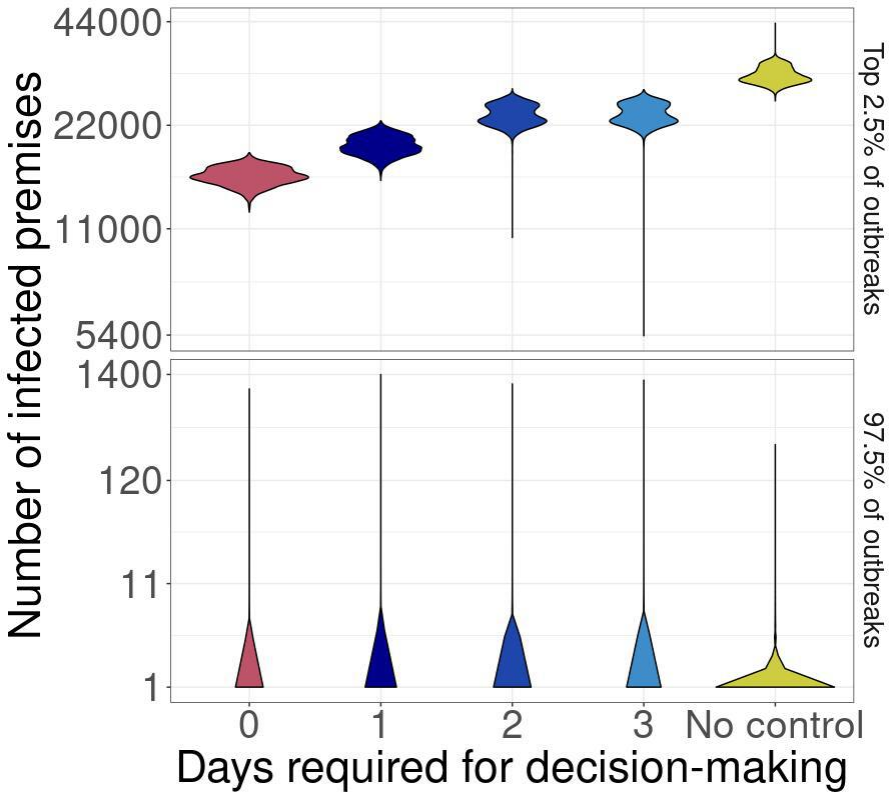
The effect of decision-making time on the number of premises infected during outbreak simulations when an MB, IP cull and DC vaccination control strategy is implemented (table 2, default strategy). Days required for decision-making (*x*-axis) is the number of days that a 10% increase in the number of new premises reports must be observed to trigger vaccination. Fixed control strategies are in red, adaptive control strategies in blue and no control in yellow. Vertical axes are faceted by outbreak size, such that 97.5% of outbreaks are in bottom panel for each outbreak metric and the largest approximately 2.5% of outbreaks are the top facet for each metric.

There was very little variation between control strategies when we varied the number of days required for decision-making, and no difference between control and no control outbreak scenarios for the smallest 97.5% of the outbreaks (figure 2). Fixed control resulted in the fewest number of IPs, even when only 1 day was required to implement the next control action in adaptive control during the largest outbreaks (figure 2; electronic supplementary material, figure SB.5). For these large outbreaks, both fixed and adaptive control strategies resulted in fewer IPs than the no control strategy. There was no difference between control strategies that required 2 and 3 days to make a decision, but both resulted in more IPs than the adaptive control strategy that only required 1 day for decision-making (figure 2). Control strategies that were allowed to de-escalate after 10 days of no new reports (table 2) did not result in fewer IPs for either the majority of outbreaks or the largest 2.5% of outbreaks (electronic supplementary material, figure SB.3).

There were no remarkable differences between the geographical distribution of outbreaks when fixed and adaptive control strategies were applied (electronic supplementary material, figure SB.7). The largest outbreaks emerged when the index infection was seeded in the USDA Basin and Range region, Great Plains or Central Florida [38]. These regions generally have the most clustered and largest number of premises with greater than 1000 animals, as well as the most incoming and outgoing animal shipments [26]. Despite producing the most severe outbreaks, outbreak metrics for these regions are still highly bi-modal, such that the median number of IPs for most counties in those regions is one across all outbreak scenarios (electronic supplementary material, figure SB.7). Number of infected counties and outbreak duration exhibited similar spatial patterns. Similarly, the geographical distribution of the proportion of infections attributable to local transmission was not noticeably different between outbreaks with adaptive and fixed control strategies for the vast majority of the US.

### 3.3. Cost analysis

The cost analysis considered the vast majority of outbreaks, despite only considering outbreaks that did not reach 365 days. Between 97.7% and 99.9% of all outbreaks, regardless of control strategy, ended prior to 365 days (electronic supplementary material, table SB.5). The per cent of outbreaks with fixed control strategies is 99.9% that ended prior to reaching 365 days while outbreaks with adaptive control strategies ended between 98.0% and 98.8% of the time. Over 97% of outbreaks faded out when no control was applied (electronic supplementary material, table SB.5).

**Figure 3.**
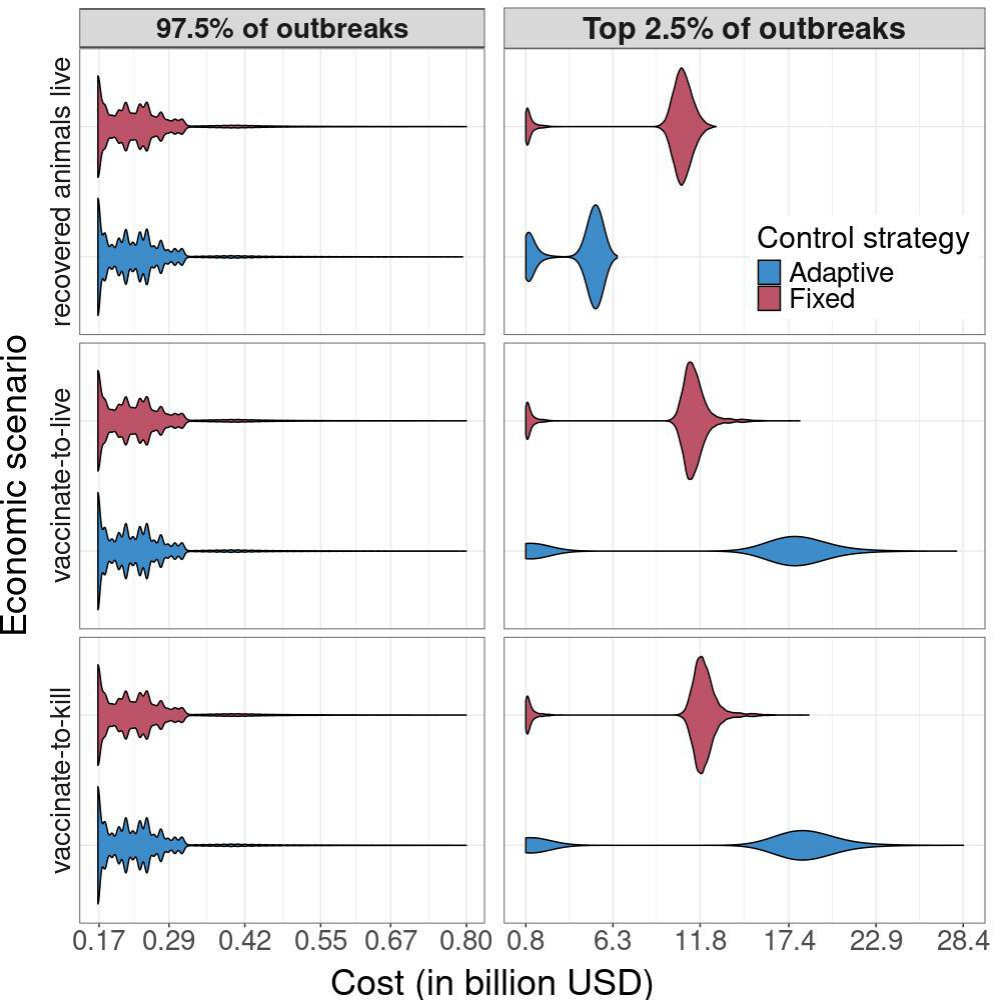
The cost of FMD outbreaks for three different economic scenarios when fixed (red) and adaptive (blue) MB, IP cull and DC vaccination control strategies are used (table 2, default strategy). The top panel is the cost when the outbreak ends and does not include the cost of culling animals that were infected, but not culled during the outbreak (equation 2.1). The middle panel assumes every infected animal will be culled, but vaccinated animals are not culled (vaccinate-to-live, equation 2.2). The bottom panel includes the cost of culling both infected and vaccinated animals (vaccinate-to-kill, equation 2.3).

Consistent with other outbreak metrics, fixed and adaptive control strategies cost the same for 97.5% of outbreaks (figure 3). For the worst 2.5% of outbreaks that faded out prior to reaching 365 days, the cost of the outbreak was dependent upon whether the cost of culling all recovered or vaccinated animals was incorporated into the cost equation (figure 3). If culling recovered or vaccinated animals was not included in the cost estimate equation 2.1, adaptive control cost less than the fixed control strategy (figure 3). However, when recovered and vaccinated animals were incorporated into the cost calculation (equation 2.2 and (2.3)), fixed control strategies cost less (figure 3). Adaptive control strategies that allowed for the de-escalation of control did not cost less than, and sometimes cost more, than strategies that did not allow for de-escalation (electronic supplementary material, figure SB.3).

**Figure 4.**
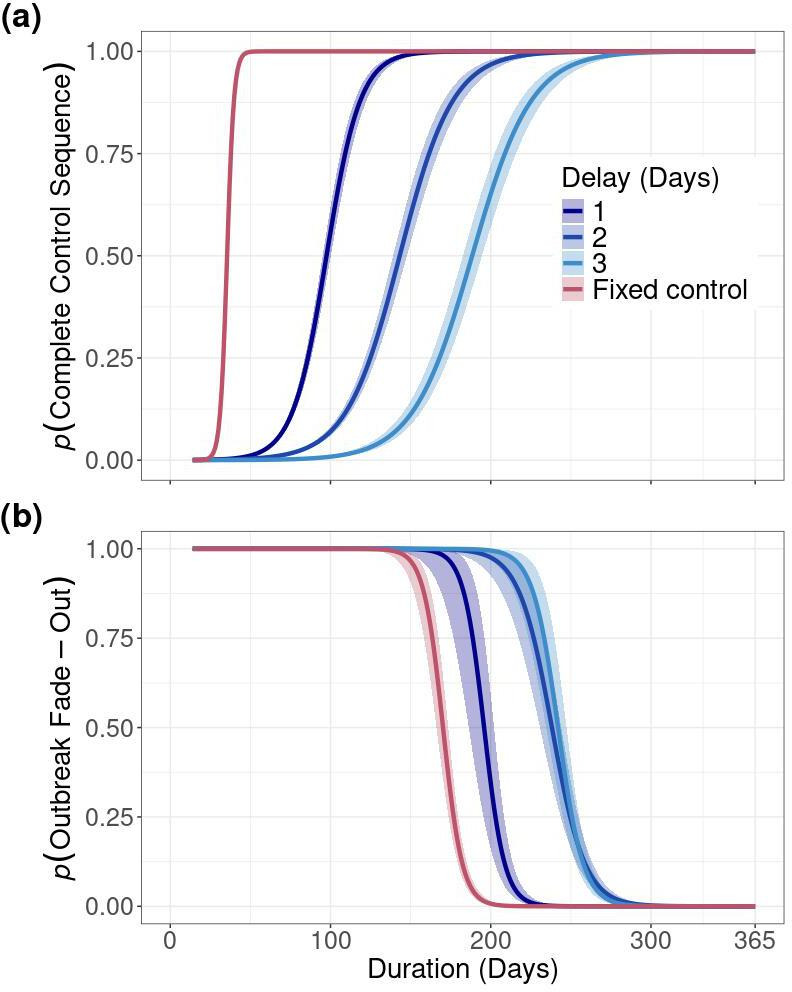
The predicted probability that (a) a control strategy is completed and (b) outbreaks fade out based on the duration of an outbreak when fixed and adaptive MB, IP cull and DC vaccination control strategies are implemented (table 2, default strategy). Solid lines are the mean probabilities and ribbons represent approximately 95% confidence intervals. We define fade-out as an outbreak spreading beyond the index infection and ending prior to infecting 5000 premises and reaching 365 days. Adaptive control strategies are blue and fixed control is in red. Probabilities are derived from logistic regression.

Fixed control strategy sequences are more likely to complete and result in an outbreak fading out than adaptive control strategies if FMD spreads beyond the initial index infection. The probability that adaptive control strategy sequences would complete during an outbreak did not reach one until 150–300 days had passed, whereas the probability reached one for a fixed control strategy by the 50th day (figure 4a). The variation around the probability a given control strategy is completed increases with the number of days required for decision-making (figure 4a). The fixed control strategy exhibited very little to no variation (electronic supplementary material, table SB.3).

The predicted probability that an outbreak fades out prior to reaching 5000 IPs and before 365 days was higher for fixed control strategies than adaptive control strategies beginning around 100 days (figure 4b). This difference grows from 100 to 364 days (electronic supplementary material, table SB.4). The predicted probability of fade-out is higher for adaptive control strategies that require one day for decision-making, rather than two or three.

### 3.4. Sensitivity analysis

**Figure 5.**
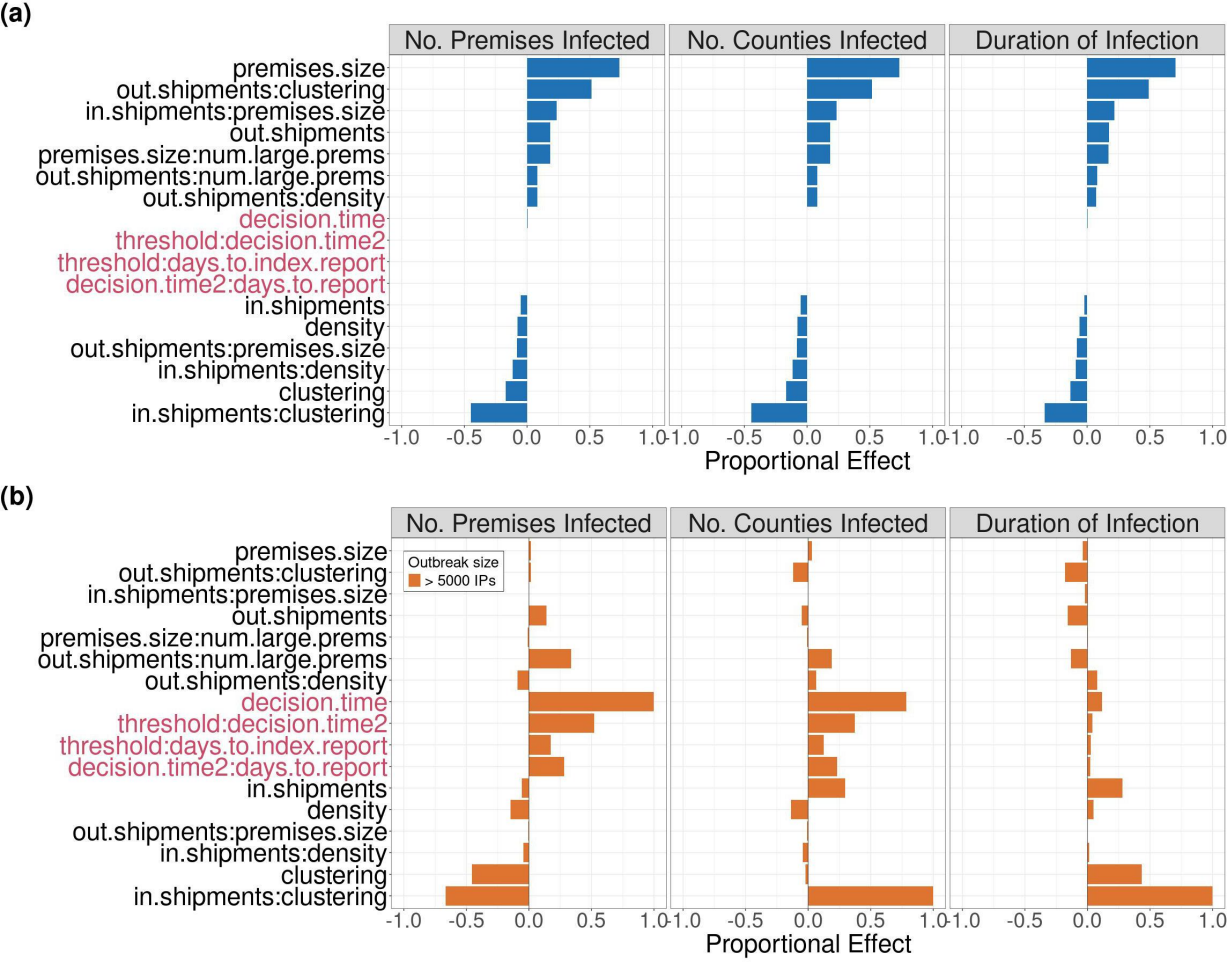
Sensitivity analysis showing the proportional effect of county demography and adaptive control strategy parameters on disease outbreak metrics when state-level movement bans, IP culling, DC vaccination is used. Adaptive control strategy parameters shown in red and county demography in black. (a) The proportional effect of each parameter when we consider all outbreaks in the regression model is shown and the proportional effect sizes in (b) are from a regression model that only considered the subset of outbreaks that infected more than 5000 premises (take-off). Results only show parameters with an effect size > 0.1 that have a significant effect on outbreak metrics (*p* < 0.05). The number two (2) following a parameter name indicates that parameter controlled the second step of the control strategy. Adaptive control parameter descriptions can be found in table 2.

The results of this sensitivity analysis revealed how sensitive our outbreak metrics were to changes in model parameters. Specifically, we evaluated the effect of adaptive control strategy parameters and demographic attributes of seed counties on outbreak metrics. Proportional effect sizes of parameters included in the sensitivity analysis were consistent across all three outbreak metrics. Attributes related to seed county demography such as index premises size, out-shipments, and pairwise interactions between out-shipments, clustering, and density were strongly positively associated with outbreak metrics. The number of large premises in a county was also strongly positively associated with outbreak metrics when interacting with seed premises size and out-shipments. Other county attributes were weakly or negatively associated with the outbreak metrics (figure 5a). All adaptive control parameters had a very weak effect on outbreak metrics. Of the reporting delays included in the analysis, the index case reporting time had a significant effect. Both of these effect sizes were also very small (figure 5a; electronic supplementary material, figure SB.8). In-shipment and index premises size were negatively correlated with outbreak metrics when they interacted with density.

Outbreak metrics were only highly sensitive to adaptive control strategy parameters for outbreaks that infected more than 5000 premises (figure 5b). Adaptive control strategy parameters were positively associated with outbreak metrics for these very large outbreaks. Changes to decision-making time and index case reporting time had the largest effect on outbreak metrics (figure 5b; electronic supplementary material, figure SB.9). However, adaptive control strategy parameters had very little effect on outbreak duration for these large outbreaks. In-shipments and clustering were the only county attributes with a strong effect on outbreak metrics for outbreaks that infected more than 5000 premises (figure 5b). For the same outbreaks, although clustering and in-shipments were very strongly positively associated with outbreak duration and the number of infected counties, they showed a weaker negative association with the number of IPs (figure 5b). Outbreak metrics were much more sensitive to demographic attributes of seed counties for outbreaks that faded-out prior to infecting 5000 premises (electronic supplementary material, figure SB.10).

## 4. Discussion

There is a strong interest in developing optimal control strategies for any given infectious disease outbreak to minimize economic losses. Unfortunately, generating an optimal control strategy for all possible future outbreak scenarios is currently a computationally intractable task. To address this, recent work has highlighted how using real-time information during an outbreak to adjust control actions can reduce an outbreak’s impact [16,23,39–41]. Although both national and international health organizations have adopted adaptive approaches to controlling infectious disease outbreaks in humans and animals [6–10], few studies have compared the impact of adaptive control strategies on outbreak outcomes against fixed control strategies. Allowing control actions to be dynamically added and removed from a control strategy and incorporating decision-making time into this process allowed us to more realistically model the process of responding to an infectious disease outbreak.

Differences between control strategies were only apparent for the largest 2.5% of outbreaks that spread to multiple premises. Given that control parameters in our sensitivity analysis had a very small effect on the size of all outbreaks (figure 5a) and were only positively associated with outbreak metrics for the largest outbreaks (figure 5b), control strategies—adaptive or not—seem to be merely preventing the largest outbreaks from becoming even larger. A previous study by this group that compared the performance of several fixed control strategies suggested that case reporting times and resource constraints render these largest outbreaks uncontrollable because they result in control trailing too far behind outbreak spread to end the outbreak [26]. Instead, county attributes like seed premises size, the number of large premises in a county and degree of premises clustering within a county are stronger drivers of whether or not an outbreak will remain small. All of these attributes have been shown to be strong drivers of outbreak outcomes and risk of infection in both the US and the UK [22,25,26,33]. Interestingly, however, IP cull DC vaccination control strategies were not improved by prioritizing large premises for control even though large premises can be more infectious [42] and drive onward transmission [43]. On the other hand, the IP cull with a 3 km ring vaccination control strategy was improved by prioritizing vaccination of premises by their distance to an infected farm, which has previously been shown to be an effective strategy [5].

When adaptive control is applied, delays associated with decision-making only exacerbate the time between becoming infectious and targeting that premises for control, explaining why adaptive control strategies result in worse large outbreaks (figure 2; electronic supplementary material, figure SB.5). Analyses of the 2001 FMD outbreak in the UK demonstrated that sudden increased demand for control resources, by identifying a very large IPs or identifying many IPs all at once, can max out local carcass disposal capacity and cause further culling delays [4,22]. Therefore, delaying the onset of an entire control strategy may not only mean that control further lags behind the spread of an infection, but it could harm the operational capacity of an outbreak response later on. These delays may also result in an increased risk of onward transmission because infected and at-risk animals become more likely to transmit the infection the longer they remain on premises while managers wait for the resources to remove them [4,22]. Current resource constraints mean a special emphasis should be placed on reducing reporting and decision-making delays to reduce the chance of making a large outbreak worse if an adaptive control strategy is adopted. Our sensitivity analysis provides further support for this recommendation as well. The relatively large effect of decision-making and case reporting times on the size of the largest outbreaks suggests that decreasing decision-making and case reporting times may be the most effective way to minimize the impact of large outbreaks (figure 5b).

In an ideal world, managers would have ample decision-making time to consider trade-offs associated with each choice made. Our results suggest that reducing time delays and resource constraints during other aspects of the control process would be necessary in order to provide this additional time for decision-making. In the absence of being able to change time delays and resource capacity, adaptive control strategies may still be suitable for less transmissible infections, like bovine tuberculosis (bTB) [44]. Less transmissible infections, like bTB, may afford managers more time decide upon a control strategy because they will not spread within or between premises as quickly. A slower spreading infection would also be less likely to max out resources and cause further delays later on in the outbreak response process. Adaptive control may also remain a feasible option if implemented on a context-dependent basis, such that its implementation is conditional on where an outbreak is seeded. This application would mean adaptive control is only used if outbreaks are seeded in counties or regions that do not pose a substantial risk for producing large outbreaks. For these reasons, adaptive control strategies may still be capable of minimizing socioeconomic harm while effectively controlling outbreaks of less transmissible infections or in lower risk populations.

One of the primary motivations for implementing a more conservative control strategy early on in an outbreak are the potential economic benefits. Our analysis suggests that these potential benefits may be outweighed by the substantial additional cost accrued by adaptive control strategies during the most severe outbreaks. Adaptive control strategies did not cost less than their fixed control counterparts even for the smallest outbreaks where we would expect to see the largest economic benefit of adaptive control. This means that adaptive control imposed a significant cost by under-reacting to an outbreak, but there was no additional cost for overreacting with fixed control. Interestingly, adaptive control was not less expensive even when control was allowed to de-escalate after 10 days of no new reports. Adaptive control strategies were only less costly when only short-term costs were considered, but became more expensive when the cost of culling recovered or vaccinated animals were incorporated into the calculation (figure 3). Adaptive control costs less when recovered animals are allowed to live in part owing to fewer animals being culled during and after the outbreak (electronic supplementary material, table SB.6). Adaptive control strategies then possess an animal welfare advantage, which may be an important consideration for policymakers and managers when deciding upon which control strategy to use.

Although our cost analysis was coarse and does not consider many additional costs [33,34], we show that even in the event of a best-case scenario, where the World Organization for Animal Health does not require all vaccinated animals to be culled after an outbreak ends, (figure 3), adaptive control still costs more money [7,35]. We also do not consider the arguably most costly element of an FMD outbreak, which is the additional time after the completion of a simulation needed to complete all control responses and move back to an FMD status that would reduce or remove trade restrictions. Given that more adaptive control simulations hit the 365 day cut-off, we would only expect that the cost difference between fixed and adaptive control to grow if this cost were incorporated. Finally, we only considered the cost of the early and explosive phases of outbreaks, where the ability to dynamically apply and remove control may be less consequential. Adaptive control may then provide a financial benefit later in the outbreak when control can be backed-off and reapplied depending upon how the outbreak’s tail behaves with less risk of allowing it to explode.

Ultimately, the goal of an adaptive control strategy would be to maximize a manager’s ability to control an outbreak while minimizing socioeconomic harm. One way to achieve this is to treat the control of an infectious disease as an optimization problem, where the cost and benefit of each control action could be considered repeatedly throughout the simulation. Such an approach may be able to achieve a more successful and less costly adaptive control strategy, but would probably require realism to be compromised for computational tractability [45]. Our ability to identify more optimal adaptive control strategies may have been limited by the fact that we only explore the most standard control strategies found in the literature. Despite this, the control strategies that performed the best remained the same regardless of whether they were implemented as adaptive or fixed control. This suggests that changing the timing of control’s onset affects its efficacy, but ultimately not which control strategies are predicted to be the best. Additionally, we consider several delays as they relate to controlling and decision-making, but do not consider how delays and uncertainty associated with diagnostic tests impact outbreaks. These additional delays are known to affect outbreak outcomes in a variety of disease systems [18,46–48]. Our decision to consider cattle-only outbreak scenarios ignores large populations of susceptible animals that, if considered, could alter a county’s demographic landscape, and therefore potentially impact transmission dynamics [49]. Given the results presented above, considering other susceptible animal populations, such as sheep, goats and pigs [50,51] would probably result in larger outbreaks and more constrained resources. Even so, cattle-only outbreak scenarios remain a reasonable choice for the study here because the majority of FMD outbreaks in non-endemic countries, like the US, are identified in cattle first and remain in cattle populations for the outbreak’s duration [52,53].

We have systematically compared the performance of several plausible adaptive and fixed control strategies to understand the potential benefits and challenges associated with minimizing economic harm while controlling an infectious disease outbreak. We warn against using our results as policy recommendations as the optimal disease control strategy is probably highly dependent upon the state of an ongoing outbreak, particularly current resource constraints [5]. Instead, this work demonstrates that any additional delays in implementing control actions contribute to producing larger and longer FMD outbreaks. These findings highlight the importance of outbreak scenario modelling efforts and developing outbreak response strategies *a priori* to minimize the time required to respond to a novel outbreak. While FMD specific, these results may provide some insight into how adaptive control strategies for other highly transmissible viral infections in livestock, such as African swine fever or highly pathogenic avian influenza, might fare during an epidemic [54,55].

## Data Availability

Data and relevant code for this research work are stored in GitHub: https://github.com/webblabb/usdos and have been archived within the Zenodo repository [56]. At the ‘U.S. Animal Movement Model and Disease Outbreak Simulation’ website (https://webblabb.github.io/usammusdos/index.html), there is a user manual as well as a link to the public GitHub repository which houses the code. Supplementary material is available online [57].

## References

[1] Kiszewski A. 2007 Estimated global resources needed to attain international malaria control goals. Bull. World Health Organ. **85**, 623–630. (10.2471/blt.06.039529) 17768521 PMC2636386

[2] Worby CJ, Chang HH. 2020 Face mask use in the general population and optimal resource allocation during the COVID-19 pandemic. Nat. Commun. **11**, 4049. (10.1038/s41467-020-17922-x) 32792562 PMC7426871

[3] Gostin LO, Friedman EA. 2015 A retrospective and prospective analysis of the West African Ebola virus disease epidemic: robust national health systems at the foundation and an empowered WHO at the apex. Lancet **385**, 1902–1909. (10.1016/s0140-6736(15)60644-4) 25987158

[4] de Klerk PF. 2002 Carcass disposal: lessons from The Netherlands after the foot and mouth disease outbreak of 2001. Rev. Sci. Et Tech. **21**, 789–796. (10.20506/rst.21.3.1376) 12523715

[5] Tildesley MJ, Savill NJ, Shaw DJ, Deardon R, Brooks SP, Woolhouse MEJ, Grenfell BT, Keeling MJ. 2006 Optimal reactive vaccination strategies for a foot-and-mouth outbreak in the UK. Nature **440**, 83–86. (10.1038/nature04324) 16511494

[6] World Health Organization. 2017 Pandemic influenza risk management: a who guide to inform and harmonize national and international pandemic preparedness and response. p. 62. World Health Organization. See https://iris.who.int/handle/10665/259893.

[7] USDA-APHIS. 2020 Foot-and-mouth disease: the Red Book. United States Department of Agriculture. See https://www.aphis.usda.gov/sites/default/files/fmd_responseplan.pdf.

[8] Holloway R, Rasmussen S, Zaza S, Cox NJ, Jernigan DB. 2014 Updated preparedness and response framework for influenza pandemics. Centers for Disease Control and Prevention. See https://www.cdc.gov/mmwr/pdf/rr/rr6306.pdf. 25254666

[9] PHE. 2014 Pandemic influenza response plan. Public Health England. See https://covid19.public-inquiry.uk/wp-content/uploads/2023/07/22160242/INQ000090387.pdf.

[10] AGDoH. 2019 Australian health management plan for pandemic influenza (AHMPPI). Austrailian Government Department of Health. See https://www.health.gov.au/sites/default/files/documents/2022/05/australian-health-management-plan-for-pandemic-influenza-ahmppi.pdf. 25980184

[11] Haydon DT, Kao RR, Kitching RP. 2004 The UK foot-and-mouth disease outbreak - the aftermath. Nat. Rev. Microbiol. **2**, 675–681. (10.1038/nrmicro960) 15263902

[12] Nicoll A. 2012 Développer la préparation en cas de pandémie en Europe au 21 siècle: expérience, évolution et prochaines étapes. Bull. World Health **90**, 311–317. (10.2471/BLT.11.097972) PMC3324872 22511829

[13] Parada LV. 2011 Life lessons. Nature New Biol. **480**, S11–S13. (10.1038/480S11a) PMC7095214 22158294

[14] Smith KF, Goldberg M, Rosenthal S, Carlson L, Chen J, Chen C, Ramachandran S. 2014 Global rise in human infectious disease outbreaks. J. R. Soc. Interface **11**, 20140950. (10.1098/rsif.2014.0950) 25401184 PMC4223919

[15] Marani M, Katul GG, Pan WK, Parolari AJ. 2021 Intensity and frequency of extreme novel epidemics. Proc. Natl Acad. Sci. USA **118**, 1–4. (10.1073/pnas.2105482118) PMC8536331 34426498

[16] Shea K, Tildesley MJ, Runge MC, Fonnesbeck CJ, Ferrari MJ. 2014 Adaptive management and the value of information: learning via intervention in epidemiology. PLoS Biol. **12**, e1001970. (10.1371/journal.pbio.1001970) 25333371 PMC4204804

[17] Tao Y, Shea K, Ferrari M. 2018 Logistical constraints lead to an intermediate optimum in outbreak response vaccination. PLOS Comput. Biol. **14**, e1006161. (10.1371/journal.pcbi.1006161) 29791432 PMC5988332

[18] Brook CE, Northrup GR, Ehrenberg AJ, Doudna JA, Boots M. 2021 Optimizing COVID-19 control with asymptomatic surveillance testing in a university environment. Epidemics **37**, 100527. (10.1016/j.epidem.2021.100527) 34814094 PMC8591900

[19] Larremore DB, Wilder B, Lester E, Shehata S, Burke JM, Hay JA, Tambe M, Mina MJ, Parker R. 2021 Test sensitivity is secondary to frequency and turnaround time for COVID-19 screening. Sci. Adv **7**, eabd5393. (10.1126/sciadv.abd5393) 33219112 PMC7775777

[20] Mina MJ, Parker R, Larremore DB. 2020 Rethinking COVID-19 test sensitivity — a strategy for containment. N. Engl. J. Med. **383**, e120. (10.1056/nejmp2025631) 32997903

[21] Bergstrom T, Bergstrom CT, Li H. 2020 Frequency and accuracy of proactive testing for COVID-19. medRxiv. (10.1101/2020.09.05.20188839)

[22] Tao Y, Probert WJM, Shea K, Runge MC, Lafferty K, Tildesley M, Ferrari M. 2021 Causes of delayed outbreak responses and their impacts on epidemic spread. J. R. Soc. Interface **18**, 20200933. (10.1098/rsif.2020.0933) 33653111 PMC8086880

[23] Probert WJM *et al*. 2018 Real-time decision-making during emergency disease outbreaks. PLOS Comput. Biol. **14**, e1006202. (10.1371/journal.pcbi.1006202) 30040815 PMC6075790

[24] Li SL, Bjørnstad ON, Ferrari MJ, Mummah R, Runge MC, Fonnesbeck CJ, Tildesley MJ, Probert WJM, Shea K. 2017 Essential information: uncertainty and optimal control of Ebola outbreaks. Proc. Natl Acad. Sci. USA **114**, 5659–5664. (10.1073/pnas.1617482114) 28507121 PMC5465899

[25] Gilbertson K *et al*. 2022 The importance of livestock demography and infrastructure in driving foot and mouth disease dynamics. Life **12**, 1604. (10.3390/life12101604) 36295038 PMC9605081

[26] Tsao K *et al*. 2020 Effects of regional differences and demography in modelling foot-and-mouth disease in cattle at the national scale. Interface Focus **10**, 20190054. (10.1098/rsfs.2019.0054) 31897292 PMC6936011

[27] Buhnerkempe MG, Grear DA, Portacci K, Miller RS, Lombard JE, Webb CT. 2013 A national-scale picture of U.S. cattle movements obtained from Interstate Certificate of Veterinary Inspection data. Prev. Vet. Med. **112**, 318–329. (10.1016/j.prevetmed.2013.08.002) 24035137

[28] Lindström T, Grear DA, Buhnerkempe M, Webb CT, Miller RS, Portacci K, Wennergren U. 2013 A Bayesian approach for modeling cattle movements in the United States: scaling up a partially observed network. PLoS One **8**, e53432. (10.1371/journal.pone.0053432) 23308223 PMC3537632

[29] Sellman S, Beck-Johnson LM, Hallman C, Miller RS, Bonner KAO, Portacci K, Webb CT, Lindström T. 2022 Modeling U.S. cattle movements until the cows come home: who ships to whom and how many? Comput. Electron. Agric. **203**, 107483. (10.1016/j.compag.2022.107483)

[30] Burdett CL, Kraus BR, Garza SJ, Miller RS, Bjork KE. 2015 Simulating the distribution of individual livestock farms and their populations in the United States: an example using domestic swine (Sus scrofa domesticus) farms. PLoS ONE **10**, e0140338. (10.1371/journal.pone.0140338) 26571497 PMC4646625

[31] National Agricultural Statistics Service, Agriculture Department. 2012 2012 Census of agriculture: volume 1, part 51, geographic area series. United States summary and state data, part 51, United States summary and state data, 1, geographic area series*.* Agriculture Department. See https://www.govinfo.gov/app/details/GOVPUB-A92-PURL-gpo44347.

[32] Runge MC, Converse SJ, Lyons JE. 2011 Which uncertainty? Using expert elicitation and expected value of information to design an adaptive program. Biol. Conserv. **144**, 1214–1223. (10.1016/j.biocon.2010.12.020)

[33] Schoenbaum MA, Terry Disney W. 2003 Modeling alternative mitigation strategies for a hypothetical outbreak of foot-and-mouth disease in the United States. Prev. Vet. Med. **58**, 25–52. (10.1016/s0167-5877(03)00004-7) 12628769

[34] Knight-Jones TJD, Rushton J. 2013 The economic impacts of foot and mouth disease – what are they, how big are they and where do they occur? Prev. Vet. Med. **112**, 161–173. (10.1016/j.prevetmed.2013.07.013) 23958457 PMC3989032

[35] World Organisation for Animal Health (WOAH). 2022 Terrestrial animal health code. WOAH. See https://www.woah.org/en/what-we-do/standards/codes-and-manuals/#/?tab=0.

[36] Pang Z, O’Neill Z. 2019 A comparison study of various sensitivity analysis methods in building applications. IBPSA 4498–4406. (10.26868/25222708.2019.210209)

[37] Blower SM, Dowlatabadi H, Dowlatabadit H. 1994 Sensitivity and uncertainty analysis of complex models of disease transmission: an HIV model, as an example. International Statistical Review **3**, 229–243. (10.2307/1403510)

[38] Vaiknoras K, Hubbs T. 2023 Characteristics and trends of U.S. soybean production practices, costs, and returns since 2002. U. S. Dep. Agric. Econ. Res. Serv **9**, 1572255. (10.32747/2023.8023698.ers)

[39] Pepin KM, Brown VR, Yang A, Beasley JC, Boughton R, VerCauteren KC, Miller RS, Bevins SN. 2022 Optimising response to an introduction of African swine fever in wild pigs. Transbound. Emerg. Dis. **69**, e3111–e3127. (10.1111/tbed.14668) 35881004

[40] Miller RS, Pepin KM. 2019 Board invited review: prospects for improving management of animal disease introductions using disease-dynamic models. J. Anim. Sci. **97**, 2291–2307. (10.1093/jas/skz125) 30976799 PMC6541823

[41] Shearer FM, Moss R, McVernon J, Ross JV, McCaw JM. 2020 Infectious disease pandemic planning and response: incorporating decision analysis. PLOS Med. **17**, e1003018. (10.1371/journal.pmed.1003018) 31917786 PMC6952100

[42] Beck-Johnson LM, Gorsich EE, Hallman C, Tildesley MJ, Miller RS, Webb CT. 2023 An exploration of within-herd dynamics of a transboundary livestock disease: a foot and mouth disease case study. Epidemics **42**, 100668. (10.1016/j.epidem.2023.100668) 36696830

[43] Keeling MJ. 2005 Models of foot-and-mouth disease. Proc. R. Soc. B **272**, 1195–1202. (10.1098/rspb.2004.3046) PMC1564112 16024382

[44] Álvarez J, Bezos J, de la Cruz ML, Casal C, Romero B, Domínguez L, de Juan L, Pérez A. 2014 Bovine tuberculosis: within-herd transmission models to support and direct the decision-making process. Res. Vet. Sci. **97**, S61–S68. (10.1016/j.rvsc.2014.04.009) 24875061

[45] Probert WJM, Lakkur S, Fonnesbeck CJ, Shea K, Runge MC, Tildesley MJ, Ferrari MJ. 2019 Context matters: using reinforcement learning to develop human-readable, state-dependent outbreak response policies. Phil. Trans. R. Soc. B **374**, 20180277. (10.1098/rstb.2018.0277) 31104604 PMC6558555

[46] Carpenter TE, O’Brien JM, Hagerman AD, McCarl BA. 2011 Epidemic and economic impacts of delayed detection of foot-and-mouth disease: a case study of a simulated outbreak in California. J. Vet. Diagn. Investig. **23**, 26–33. (10.1177/104063871102300104) 21217024

[47] Rong X, Yang L, Chu H, Fan M. 2020 Effect of delay in diagnosis on transmission of COVID-19. Math. Biosci. Eng. **17**, 2725–2740. (10.3934/mbe.2020149) 32233563

[48] East IJ, Martin PAJ, Langstaff I, Iglesias RM, Sergeant ESG, Garner MG. 2016 Assessing the delay to detection and the size of the outbreak at the time of detection of incursions of foot and mouth disease in Australia. Prev. Vet. Med. **123**, 1–11. (10.1016/j.prevetmed.2015.12.005) 26718055

[49] Kinsley AC, VanderWaal K, Craft ME, Morrison RB, Perez AM. 2018 Managing complexity: simplifying assumptions of foot-and-mouth disease models for swine. Transbound. Emerg. Dis. **65**, 1307–1317. (10.1111/tbed.12880) 29687629

[50] Orsel K, Bouma A, Dekker A, Stegeman JA, de Jong MCM. 2009 Foot and mouth disease virus transmission during the incubation period of the disease in piglets, lambs, calves, and dairy cows. Prev. Vet. Med. **88**, 158–163. (10.1016/j.prevetmed.2008.09.001) 18929417

[51] Kitching RP, Hughes GJ. 2002 Clinical variation in foot and mouth disease: sheep and goats. Rev. Sci. Tech. OIE. **21**, 505–512. (10.20506/rst.21.3.1342) 12523691

[52] McLaws M, Ribble C. 2007 Description of recent foot and mouth disease outbreaks in nonendemic areas: exploring the relationship between early detection and epidemic size. Can. Vet. J. **48**, 1051–1062. 17987967 PMC1978293

[53] Beck-Johnson *et al*. 2013 An exploration of within-herd dynamics of a transboundary livestock disease: a foot and mouth disease case study. 10.1016/j.epidem.2023.100668 36696830

[54] Hill EM *et al*. 2018 The impact of surveillance and control on highly pathogenic avian influenza outbreaks in poultry in Dhaka Division, Bangladesh. PLoS Comput. Biol. **14**, e1006439. (10.1371/journal.pcbi.1006439) 30212472 PMC6155559

[55] O’Neill X, White A, Ruiz-Fons F, Gortázar C. 2020 Modelling the transmission and persistence of African swine fever in wild boar in contrasting European scenarios. Sci. Rep. **10**, 5895. (10.1038/s41598-020-62736-y) 32246098 PMC7125206

[56] Beck-Johnson L, Leach C, Bonner KO. 2025 Webblabb/usdos: V2.2.1. Zenodo (10.5281/zenodo.15788077)

[57] Smith S, Webb CT, Sellman S, Lindström T, Beck-Johnson LM. 2025 Supplementary material from: Potential benefits of adaptive control strategies are outweighed by costs of infrequent, but dramatically larger disease outbreaks. Figshare. (10.6084/m9.figshare.c.7948814) PMC12368609 40852133

